# Quantitative Assessment of Tissue Perfusion in Hepatocellular Carcinoma Using Perflubutane Dynamic Contrast-Enhanced Ultrasonography: A Preliminary Study

**DOI:** 10.3390/diagnostics5020210

**Published:** 2015-05-20

**Authors:** Naoki Ohno, Tosiaki Miyati, Makiko Yamashita, Mayu Narikawa

**Affiliations:** 1 Faculty of Health Sciences, Institute of Medical, Pharmaceutical and Health Sciences, Kanazawa University, 5-11-80, Kodatsuno, Kanazawa, Ishikawa 9200942, Japan; E-Mail: nohno@med.kanazawa-u.ac.jp; 2 Department of Radiology, Tonami General Hospital, 16-1, Shintomicho, Tonami, Toyama 9391395, Japan; E-Mails: karkulaiset.0323@gmail.com (M.Y.); mayu.narikawa@med.tonami.toyama.jp (M.N.)

**Keywords:** contrast-enhanced ultrasonography (CEUS), perflubutane, low mechanical index, hepatocellular carcinoma (HCC), perfusion index

## Abstract

Our purpose in this study was to assess the relationship between contrast signal intensity (CI) and concentration of perflubutane microbubbles in a phantom experiment, and to examine the feasibility of this technique for quantitative analysis of vascularity in hepatocellular carcinoma (HCC). Microbubble solutions of the perflubutane contrast agent were prepared by mixing with purified water. We examined the relationship between CI in dB units and the concentration. Moreover, seven HCC patients were examined using real-time dynamic contrast imaging. The perfusion index was calculated from time-intensity curves generated for both HCC and surrounding liver parenchyma. We observed a linear relationship between the CI_dB_ and the concentration in the phantom study and a higher perfusion index in the HCC lesions relative to the surrounding liver parenchyma. Dynamic contrast-enhanced ultrasonography with perflubutane microbubbles, which exhibit linear and temporally stable characteristics under continuous ultrasound exposure, allows the collection of quantitative hemodynamic information regarding HCC.

## 1. Introduction

Evaluations of tissue hemodynamics are useful for assessing tissue viability, characterizing tumor properties, and predicting treatment responses to chemotherapy [1,2,3]. Although contrast-enhanced dynamic computed tomography (CT) and magnetic resonance imaging (MRI) are commonly used to assess tissue perfusion, dynamic contrast-enhanced ultrasonography (DCE-US) can also easily provide detailed information regarding tissue vascularity [4,5,6,7,8]. A mixture of galactose and palmitic acid has been widely used as a first-generation contrast agent for liver DCE-US, and the efficacy of this agent is attributable to the destruction of microbubbles caused by high sound pressure levels. Recently, second-generation contrast agents, Sulphur hexafluoride microbubbles (SonoVue), perflutren lipid microbubbles (Definity), perflutren protein microbubbles (Optison), and perflubutane microbubbles (Sonazoid), were developed to improve the diagnosis of hepatocellular carcinoma (HCC) [9,10]. These contrast agents have the advantages of a long imaging duration and strong contrast effects that allow continuous real-time imaging at a low mechanical index; these advantages are conferred by microbubbles that are more stable than those in first-generation contrast agents, which easily collapse under ultrasound pressure [11]. Unlike other second-generation contrast agents, perflubutane microbubbles are phagocytized by Kupffer cells, thereby enabling Kupffer imaging in the post-vascular phase [12]. The perflubutane microbubbles contrast agent is licensed only in Japan and South Korea, and DCE-US with the perflubutane microbubbles has been recommended in the Japanese guidelines for the management of HCC [13].

In some papers and guidelines for the contrast enhanced ultrasound of the liver, quantitative analysis of DCE-US data has been recommended for monitoring the response to antiangiogenic treatment [10,14,15]. However, the nonlinear relation between the ultrasound signal intensity and the perflubutane microbubble concentration distorts quantitative features of DCE-US. Thus, DCE-US-based assessments of tissue vascularity using perflubutane microbubbles have generally been done by visual assessments [16,17], although this technique might be useful for the early detection and differentiation of focal liver lesions and evaluations of therapeutic responses.

Contrast signal intensity (CI) has previously been used to evaluate the degree of contrast enhancement in DCE-US [18,19]. CI reflects the microbubble concentration and enables quantitative assessments of tissue vascularity, assuming that a linear relationship exists between CI and the concentration of the ultrasound contrast agent. However, to our knowledge, linearity between CI and the concentration of the perflubutane contrast agent has not yet been reported. Therefore, the aim of this study was to assess the relationship between CI and the concentration of perflubutane microbubbles in a phantom experiment and to investigate the feasibility of our quantitative method for determining tumor vascularity in HCC.

## 2. Experimental Section

### 2.1. Phantom Study

**Figure 1:**
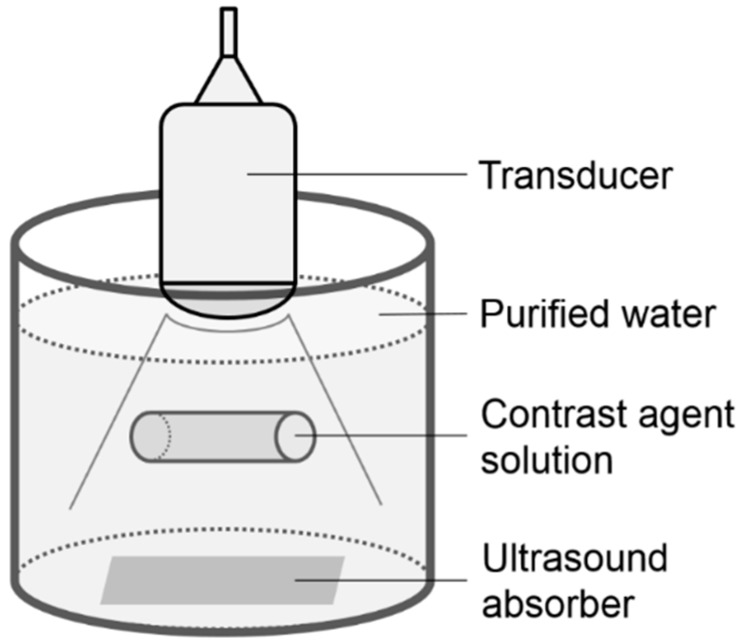
Illustration of the phantom.

**Figure 2:**
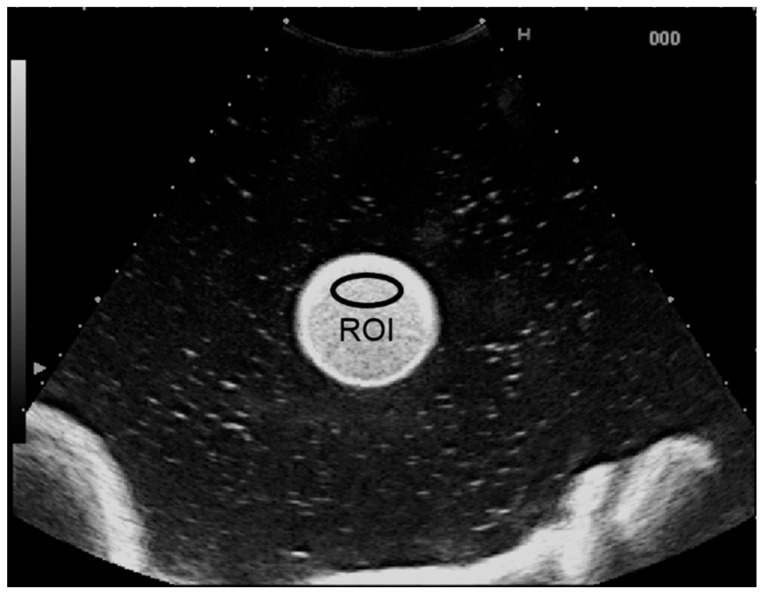
Ultrasound image of the contrast agent solution. The contrast signal intensity (CI) was measured in the region of interest (ROI) placed within the solution.

Microbubble solutions of the perflubutane contrast agent (Sonazoid; Daiichi-Sankyo, Tokyo, Japan) were prepared by mixing with purified water to final concentrations of 1.91, 3.81, 15.3, 61.0, and 244.1 μL/L. These solutions were each enclosed in a cylindrical rubber bag (diameter, 35 mm; height, 180 mm; thickness, 0.03 mm) that was attached near the center of a tank filled with purified water (Figure 1). A transducer was placed on the upper surface of the filled rubber bag. Ultrasound images of each solution were recorded for 10 s using an ultrasound system (HV900; Hitachi Medical Corporation, Tokyo, Japan) fitted with a 3.5-MHz convex-array transducer (EUP-C715) with a mechanical index of 0.17. Image analysis was performed using a brightness measurement program installed on the ultrasound system. An ellipsoidal region of interest (ROI) was placed within the solution (Figure 2), and CI in dB units was determined from the received signal using the equation 10 × log_10_CI. The contrast agent concentration was also calculated in dB as 10 × log_10_[C/C_0_], where C was the contrast agent concentration in each solution and C_0_ was the reference value of 1.91 μL/L. A linear regression analysis was used to examine the relationship between CI_dB_ and the concentration.

### 2.2. Clinical Study Subjects

The purpose and all procedures associated with our investigation were fully explained to all subjects, and the studies were conducted only after we obtained informed consent from each subject. This study was approved by the institutional review board at Tonami General Hospital. In this study, seven HCC patients (five men and two women; mean age, 74.7 ± 8.4 years) were examined. All patients underwent CT during arterial portography and CT during hepatic arteriography, and HCC diagnoses were confirmed from these imaging findings.

### 2.3. Procedure for Dynamic Contrast-Enhanced US Analysis

All patients were examined using the same ultrasound system and 3.5-MHz convex-array transducer. First, 0.015 mL/kg of perflubutane contrast agent (Sonazoid; Daiichi-Sankyo, Tokyo, Japan) was manually injected intravenously as a bolus, followed by a flush with 10 mL of saline. We initiated real-time dynamic contrast imaging with a low mechanical index (0.33) immediately after injecting the contrast agent. Breath holding was performed during the arterial phase (0–40 s after contrast injection) to avoid respiratory motion. DCE-US images for each patient were exported in digital video-clip (DICOM format) at 10 frames per second for the following offline post-processing.

In HCC nodules, ROIs were drawn manually and defined on the frame corresponding to the best identification of the lesion during the arterial phase using Image J software (NIH, Bethesda, MD, USA). ROIs in the liver parenchyma and hepatic artery were defined near the same depth as those in the HCC nodules. We determined the mean CIs within ROIs on DCE-US images from each phase. CI_dB_ was determined as 10 × log_10_CI. Time-intensity curves (TICs) were generated for both HCC and the liver parenchyma.

On the basis of the general equation for determining tissue blood flow from dynamic CT data as described by Miles [1], the perfusion index was determined as follows:

(1) $$Perfusion\;index=\frac{S_{tissue}}{arterial\;CI_{dB}}$$

where *S_tissue_* represents the maximum slope of tissue enhancement (dB per minute) and *arterial CI_dB_* represents peak arterial enhancement (dB). These parameters were calculated from TICs. The perfusion index was calculated from all phase images on a pixel-by-pixel basis, and a perfusion index map was created. All calculations were performed at MATLAB (MathWorks, Natick, MA, USA).

Perfusion indices were compared between HCC and the liver parenchyma using the Wilcoxon signed rank test. *P* < 0.05 was considered to indicate a significant difference. All statistical analyses were performed using statistical software (SPSS for Windows, version 18.0; SPSS, Chicago, IL, USA).

## 3. Results

### 3.1. Phantom Study

**Figure 3:**
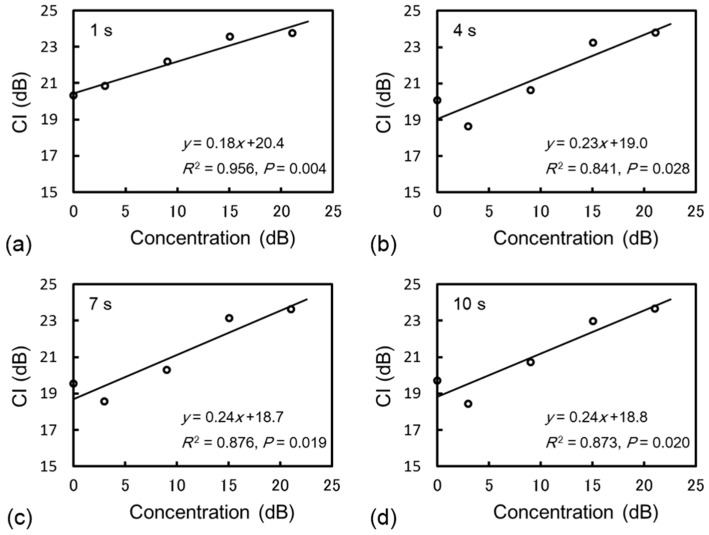
Relationship between CI and the contrast agent concentration in dB units (**a**) 1, (**b**) 4, (**c**) 7, and (**d**) 10 s after scan initiation. There was a positive correlation between CI and the concentration in dB within the actual concentration range of the agent in the human body.

Figure 3 shows the relationship between CI_dB_ and the contrast agent concentration 1, 4, 7, and 10 s after scan initiation. The regression analysis demonstrated that CI_dB_ exhibited a linear correlation with the concentration within the actual concentration range of the agent in the human body.

### 3.2. Clinical Study

**Figure 4:**
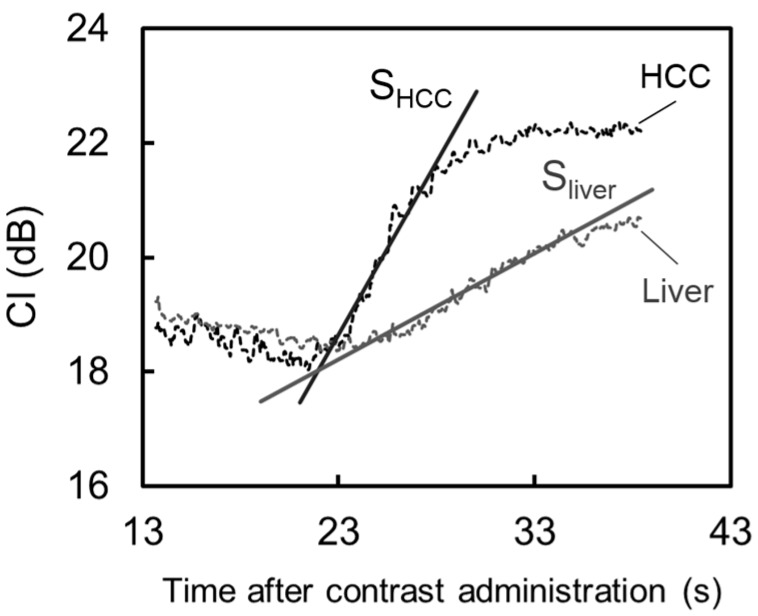
Time intensity curves at hepatocellular carcinoma (HCC) and liver parenchyma. Rapid enhancement was observed in HCC compared with the liver parenchyma. Maximum slopes of enhancement curve were determined in HCC and the liver parenchyma (S_HCC_ and S_liver_, respectively).

**Figure 5:**
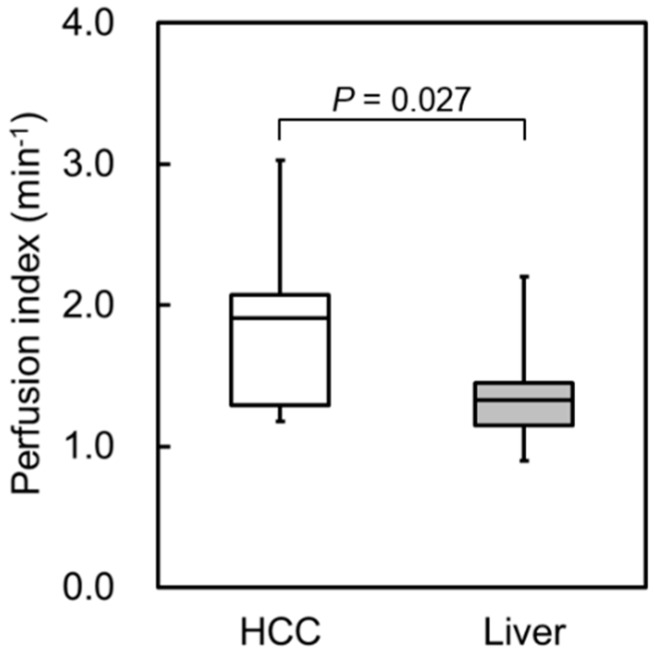
Perfusion indices in hepatocellular carcinoma (HCC) and the liver parenchyma. The HCC perfusion index was significantly higher than the liver parenchyma perfusion index.

**Figure 6:**
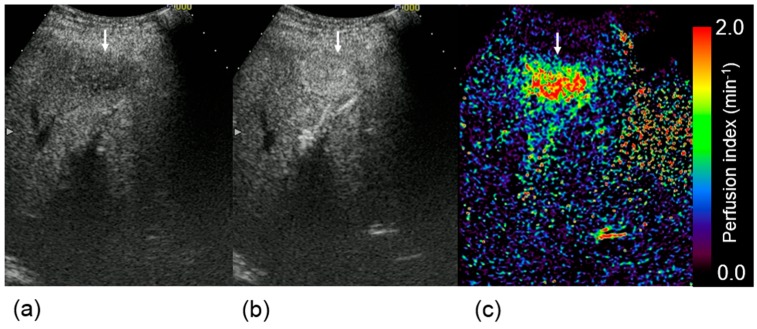
Dynamic contrast-enhanced ultrasound images obtained (**a**) before and (**b**) 30 s after contrast injection, and (**c**) a perfusion index map in the same subject. White arrows indicate hepatocellular carcinoma.

Typical TICs in HCC and the liver parenchyma are shown in Figure 4. CI_dB_ in HCC increased more rapidly during the arterial phase compared with that in the liver parenchyma. Figure 5 shows the perfusion indices in HCC and the liver parenchyma. Figure 6 presents examples of DCE-US images and perfusion index maps. The HCC perfusion index was significantly higher than the liver parenchyma perfusion index.

## 4. Discussion

To our knowledge, the quantification of tissue perfusion via DCE-US with perflubutane microbubbles has been limited to visual assessments because linearity between CI and the concentration of a perflubutane contrast agent had not been confirmed. In the present study, we therefore investigated the relationship between CI_dB_ and the concentration of perflubutane microbubbles under continuous ultrasound exposure. The results of our phantom experiment demonstrated that CI_dB_ correlated logarithmically and linearly with the concentration of perflubutane microbubbles within the actual concentration range of the contrast agent in the human body. Yamada *et al.* [18,19] reported a positive correlation between CI measured in dB and the concentration of a galactose-based contrast agent under conditions of constant applied acoustic pressure, thus allowing a quantitative assessment of tissue perfusion. Our results also demonstrated linearity between CI_dB_ and the concentration of the perflubutane contrast agent, as well as the other second generation US contrast agents. This result indicates that DCE-US with perflubutane microbubbles enables the collection of perfusion information via quantitative analysis.

Moreover, we quantitatively evaluated the vascularity in HCC lesions using CI_dB_. The perfusion index proposed by Miles [1] was used for the quantitative assessments of the dynamic US imaging data. Consequently, rapid enhancement was observed in the HCC lesions after contrast injection, and the perfusion index in the HCC region was significantly higher than that in the liver parenchyma. The rapid change in the CI_dB_ of the HCC after contrast injection, as shown in Figure 4, might reflect the status of intratumoral angiogenesis. This result suggests that the perfusion index evaluation in dynamic US enables the quantitative assessment of tumor vascularity in HCC lesions. We have also considered that this method might be more useful for evaluating the effectiveness of antiangiogenic therapies as well as diagnosing liver tumors. As our method requires no additional data and only the quantitatively analysis of DCE-US data, which is generally acquired in clinical practice, the burden upon the subjects is not increased, thereby facilitating the clinical application of this technique.

Our study had some limitations. First, because the patient population was small, we recommend that a further evaluation be undertaken with a larger sample size to confirm the efficacy of the method described in this study. Second, we did not consider the effect of portal perfusion on hepatic perfusion quantification since we could not quantify DCE-US data at the portal venous phase because of difficulty in breath holding immediately after the arterial phase scan (40 s). However, the assessment of portal perfusion is essential, especially in case of HCC that occurs on cirrhosis in the majority of cases. Thus, a further investigation with consideration of the use of respiratory gating and the effect of portal perfusion should be pursued in the future.

## 5. Conclusions

Even with some limitations, we confirmed the linearity between CI_dB_ and the concentration of perflubutane microbubbles in the phantom study and indicated the feasibility of our quantitative perfusion analysis for evaluating vascularity of HCC in the preliminary clinical study. Thus, DCE-US with perflubutane microbubbles, which exhibit linear and temporally stable characteristics under continuous ultrasound exposure, allows the collection of more detailed and quantitative hemodynamic information regarding HCC.
